# Molecular Determinants of Mouse Neurovirulence and Mosquito Infection for Western Equine Encephalitis Virus

**DOI:** 10.1371/journal.pone.0060427

**Published:** 2013-03-27

**Authors:** Eric C. Mossel, Jeremy P. Ledermann, Aaron T. Phillips, Erin M. Borland, Ann M. Powers, Ken E. Olson

**Affiliations:** 1 Arthropod-borne and Infectious Diseases Laboratory, Department of Microbiology, Immunology and Pathology, Colorado State University, Fort Collins, Colorado, United States of America; 2 Division of Vector-Borne Infectious Diseases, Centers for Disease Control, Fort Collins, Colorado, United States of America; Blood Systems Research Institute, United States of America

## Abstract

Western equine encephalitis virus (WEEV) is a naturally occurring recombinant virus derived from ancestral Sindbis and Eastern equine encephalitis viruses. We previously showed that infection by WEEV isolates McMillan (McM) and IMP-181 (IMP) results in high (∼90–100%) and low (0%) mortality, respectively, in outbred CD-1 mice when virus is delivered by either subcutaneous or aerosol routes. However, relatively little is known about specific virulence determinants of WEEV. We previously observed that IMP infected *Culex tarsalis* mosquitoes at a high rate (app. 80%) following ingestion of an infected bloodmeal but these mosquitoes were infected by McM at a much lower rate (10%). To understand the viral role in these phenotypic differences, we characterized the pathogenic phenotypes of McM/IMP chimeras. Chimeras encoding the E2 of McM on an IMP backbone (or the reciprocal) had the most significant effect on infection phenotypes in mice or mosquitoes. Furthermore, exchanging the arginine, present on IMP E2 glycoprotein at position 214, for the glutamine present at the same position on McM, ablated mouse mortality. Curiously, the reciprocal exchange did not confer mouse virulence to the IMP virus. Mosquito infectivity was also determined and significantly, one of the important loci was the same as the mouse virulence determinant identified above. Replacing either IMP E2 amino acid 181 or 214 with the corresponding McM amino acid lowered mosquito infection rates to McM-like levels. As with the mouse neurovirulence, reciprocal exchange of amino acids did not confer mosquito infectivity. The identification of WEEV E2 amino acid 214 as necessary for both IMP mosquito infectivity and McM mouse virulence indicates that they are mutually exclusive phenotypes and suggests an explanation for the lack of human or equine WEE cases even in the presence of active transmission.

## Introduction

Western equine encephalitis virus (WEEV) is an alphavirus endemic to and enzootic in North America. The virus, first isolated in 1930 by inoculation of brain material from a sick horse into a healthy one [1], is maintained in the zoonotic cycle which involves transmission from (primarily) *Culex* (*Cx.*) *tarsalis* mosquitoes to passerine birds [2]. The range of the virus principally corresponds to the geographic distribution of the mosquito vector. Periodically, humans or equines exposed to the virus via *Cx. tarsalis* or other bridge vectors have resulted in large epidemics or epizootics of encephalitis. The first equine outbreaks recorded in the 1930's affected several hundred thousand equines with mortality rates of up to 50% [1], [3]. At least 10 states in the US and 5 Canadian provinces had human and/or equine outbreaks during the 1930's–1950's. While human epidemics were not as severe as equine epizootics, the case fatality was still high ranging from 8 – 15% [3], [4]. Outbreaks continued to occur in the western parts of Canada and the US through the 1970's but the magnitude of these episodes was decreasing over time. The last documented human case of WEE in the US occurred in 1999 when a single patient in Minnesota was diagnosed with the disease [5]. Canada has seen a similar decline in WEEV presence with little or no activity detected since the 1990's.

The disappearance of WEEV disease in humans and equids suggested that WEEV had become extinct. However, the periodic isolation of the virus from mosquitoes during non-epidemic periods combined with the detection of seropositive animals in surveillance programs indicated that the virus is indeed still present in zoonotic cycles [3], [6]. Several studies using multiple North American WEEV isolates failed identify a general temporal decline in mammalian, avian, host vector, or *in vitro* virulence since the mid-1900's peak in mammalian cases [7]–[9]. Our previous work examined the virulence of several distinct strains of WEEV and demonstrated significant differences in both viremia and mortality in an outbred mouse model [10]. In contrast to earlier studies, among the specific isolates tested, virulence did appear to decline with time. In agreement with previous outbred mouse studies [11], the earliest isolate we examined, McMillan (McM), originally isolated from a human case in 1941, was lethal to CD-1 mice by 4 days post-infection (dpi) in a dose independent manner. This is in contrast to the most recent isolate in that study, IMP-181 (IMP; isolated in 2005 from mosquito pools), which induced no mortality in the same animal model system [10]. Based on those results, we hypothesized that there exists a viral genetic explanation for the differing levels of disease activity in that model. To understand the role of specific viral determinants that decrease virulence and to identify viral genetic regions or mutations responsible for this phenotypic variation, we developed a series of McM/IMP chimeric viruses and point mutants that would precisely locate which, if any, viral genetic elements regulated virulence in an animal model. Additionally, we used this same panel of viruses in *Cx. tarsalis* to evaluate the ability of various viral genetic elements to modulate infection or dissemination within the vector. Our results identify specific amino acids which impart significant changes in virulence within mice and infection within the vector mosquito.

## Methods

### Cell culture

BHK-21 (BHK; baby hamster kidney), C6/36 (*Aedes albopictus*), DF-1 (chick), and Vero E6 cells were obtained from American Type Culture Collection (Manassas, VA) or from the CDC cell culture reference section. The Neuro-2a (N2a) murine neuroblastoma cell line was a gift from Dr. Mark Zabel (Colorado State University) and was originally obtained from ATCC (catalog #CCL-131). All cells were propagated in minimal essential medium with 10% heat-inactivated fetal calf serum at 37°C (28 °C for C6/36) in a 7% CO_2_ incubator.

### Virus strains

WEEV McM, originally isolated in 1941 from the brain of an infected human, isolate was obtained from the Arbovirus Reference Collection at the Centers for Disease Control and Prevention, Fort Collins, Colorado, USA. The WEEV McM infectious clone was constructed by Dr. Thomas Welte, CSU. WEEV IMP was isolated from a *Cx. tarsalis* in 2005 in Imperial County, CA. The WEEV IMP infectious clone was constructed by Dr. Michael Anishchenko and obtained from Dr. Aaron Brault (CDC, Ft. Collins, CO). Passage history prior to cloning was as follows: WEEV McM-MP2, SMB1, V2; WEEV IMP-V2 (MP, mouse; SMB, suckling mouse brain; V, Vero cells) (Logue 2009). Seed stocks for these experiments were made by electroporation of viral RNA from infectious clones into BHK cells grown in minimal essential medium with 10% fetal calf serum. Cell culture supernatant was collected when 90% of cells showed cytopathic effect (48–72 hrs) and stored in aliquots supplemented with 20% FBS at −80°C. Virus titer was determined by plaque assay on Vero cells as previously described [10].

### Chimeric virus construction

Where possible, chimeric viruses were constructed using the existing conserved, unique restrictions sites, KpnI and AvrII, present at WEEV McM nucleotides 7576 and 9682, respectively. Construction of the chimeras receiving material not bordered by these sites and the point chimeras was based on a strategy using type IIs restriction enzymes adapted from Blakqori and Weber [12] and is outlined in detail by Saxton-Shaw et al for the generation chikungunya/o′nyong nyong chimeric viruses [13]. This method allowed the generation of chimeras and point mutants without the introduction of nonnative nucleotides to the virus.

In general, cloning was completed in a three step process. First, an amplicon was generated by PCR from the parental backbone virus clone extending from the AvrII or KpnI site toward the other (inward). The PCR primers would add a site for convenient cloning into the pUC19 polycloning site. To the other end of the amplicon, a SapI recognition site oriented inward was added so that the restriction site was the exact sequence at which the change was to occur, the chimera junction. A second restriction site was added outside the SapI site for cloning into pUC19. This amplicon was cloned into a modified pUC19 plasmid that previously had the BsmBI sites and SapI site removed. A second PCR amplicon was generated from the clone donating the fragment from the AvrII or KpnI site to the chimera junction. Outside the AvrII or KpnI site, this amplicon contained the same convenient recognition site as was added outside the SapI site in the previous amplicon. On the opposite terminus was added a SapI site oriented inward such that restriction would occur at the same site as on the first amplicon, leaving complementary overhangs. Digestion of both the previous plasmid and the second amplicon with SapI and the terminal convenient restriction site and subsequent ligation generated a plasmid cassette containing a chimeric AvrII-KpnI region. Digestion of this plasmid and the parental virus clone with AvrII and KpnI and subsequent ligation resulted in a full-length chimeric WEEV clone free of artifactual base changes introduced by the cloning process.

Generation of pair 5, which exchanges a region not bordered by AvrII or KpnI, required an additional step. Generation of the first cloning plasmid was performed with an amplicon that contained a linker enzyme site (MfeI) between the added SapI site and selected pUC19 cloning site. The second amplicon was generated from the same parent and also contained a pUC19 cloning enzyme site outside AvrII or KpnI and an MfeI site outside the SapI site. This amplicon was cloned into the first plasmid using a second pUC19 cloning enzyme site and MfeI, generating a -SapI-MfeI-SapI- motif at the point of foreign DNA insertion. The DNA to be inserted was amplified from the other parental clone, adding SapI sites to both termini, oriented inward such that the restriction sites were situated over the desired cloning junction. Digestion of this amplicon and plasmid with SapI and ligation created a cloning cassette containing the chimeric region from the AvrII to KpnI sites that were then inserted back into the parental clone as above.

Finally, the point mutants were generated by a three-step process. The region of interest, including the site to be mutated and flanking sequence to include convenient, ideally unique, cloning sites, was broken into two PCR fragments. Each PCR fragment was comprised of the sequence from the mutation site through the cloning site. The PCR primers contained SapI recognition sites oriented inward toward the mutation site and cut sites modified to leave complementary overhangs containing the desired base change. Digestion of the PCR amplicons with SapI and the relevant enzymes to restrict the opposite ends followed by a three-part ligation with a modified pUC19 plasmid yielded a plasmid construct containing the region of interest with the desired base change. Once the change was confirmed by sequence analysis, the region was digested and removed from the provisionary plasmid construct and inserted into the full-length WEEV plasmid. Full-length plasmid constructs were confirmed to have incorporated the desired change by sequence analysis.

### Virus growth curves

T-25 flasks or 24-well plates (Corning, Corning, NY) were seeded with BHK, N2a, C6/36, or DF-1 cells. When approximately 90% confluent, cell monolayers were infected with virus at low multiplicity (MOI = 0.01–0.05). At specified times, supernatant was removed from three wells or two flasks for each virus on each cell type and placed in a screw-cap cryovial at −70°C until titration by plaque assay. Titration results for each virus were compared at all time points by the two tailed t-test.

### Chick infection studies

The use of chickens was reviewed and approved by the Animal Care and Use Committee at Colorado State University (protocol #12-3418A). Care and handling of chicks was in accordance with the PHS Policy and Guide for the Care and Use of Laboratory Animals. Three day old chicks (n = 6/virus) were infected with 10^4^ plaque-forming units (pfu) of McM and IMP viruses by subcutaneous injection and monitored daily by a veterinarian for 7 days. Chicks were bled at days 1, 2, 3, and 4 dpi to determine viremia. The humane endpoint for these experiments was at the termination of the experiment (7 dpi).

### Mouse virulence studies

The use of all mice was reviewed and approved by the Animal Care and Use Committee at Colorado State University (Protocol #11-2605A). Five week-old female CD-1 mice (The Jackson Laboratory, Bar Harbor, ME or Charles River Labs, Wilmington, MA) were allowed to acclimate for 7–10 days. Viral stocks were diluted in serum-free MEM to a concentration of 2×10^4^ pfu per mL. Mice were infected in groups of 5–10, at least two replications per virus (final n = 10–15), by subcutaneous (s.c.) injection inside the left thigh with 10^3^ pfu in 50 µL. Inocula were titered by plaque assay on Vero cells to confirm dosage.

All infected and uninfected control mice were visually monitored by laboratory personnel at a minimum of two times daily. Additionally, mice were monitored once daily by Laboratory Animal Resources and results of these observations reported to the University Veterinarian. Our protocols allow continuation until clinical signs are evident. Mice exhibiting lethargy, unresponsiveness, or neurological signs (postural instability, seizures, piloerection, and/or paralysis) were euthanized by CO_2_ inhalation. The day a mouse was euthanized was considered the day of death for calculation of mean time to death (MTD). Survivorship was followed for a period of 14 days and compared among virus constructs by Fisher's exact test.

### Mosquito infections

Three to four day-old adult *Cx. tarsalis* mosquitoes were fed on a blood meal containing WEEV. The blood meal contained equal parts of virus, FBS with 10% sucrose, and goose blood (Colorado Serum CO., Boulder, CO) washed with phosphate-buffered saline and packed by centrifugation. A Hemotek feeding system (Discovery Workshops, Accrington, Lancashire, UK) was used to deliver the blood meal to the mosquitoes at 37°C. The mosquitoes were allowed to feed for 1 hour on the apparatus. The fully engorged females were separated and placed into a humidified environmental chamber (Thermo Scientific 3960, Houston, TX) and held at 28°C for 8 days until processing. Blood meal titers were determined by plaque assay and ranged from 2.0×10^5^–2.0×10^6^ pfu/mL.

After the 8 day holding period, mosquitoes were cold anesthetized and decapitated, placing the bodies into 1.7 ml tubes (Eppendorf, Hauppauge, NY). A 400 µl aliquot of Dulbecco's minimal essential medium (DMEM) (Gibco, Carlsbad, CA) supplemented with 10% fetal bovine serum (FBS), 100 U/ml of penicillin and streptomycin, 1 U/ml of fungizone and gentamycin was placed into each tube and the sample was homogenized using a pestle (Kontes, Vineland, NJ). The supernatant was clarified by filtration through a 0.2 µM syringe filter (Pall, Ann Arbor, MI) into a clean tube and placed at −70°C until use.

Virus presence was determined by pipetting 100 µL of clarified supernatant onto Vero cells in a 96-well plate. The plate was incubated at 37°C under 5% CO_2_ and monitored daily for cytopathic effect (CPE). Samples were considered virus positive if CPE was observed.

## Results

### WEEV McM E2 amino acid 214 is necessary for mouse virulence

**Figure 1:**
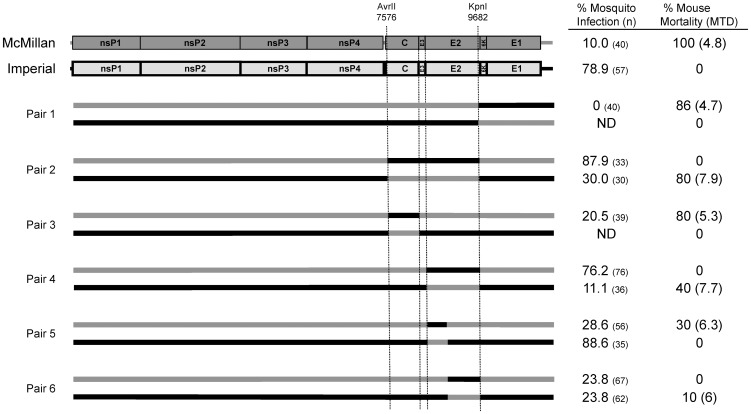
*Cx. tarsalis* infectivity and murine virulence of WEEV McM, IMP, and several chimeric viruses (gray  =  WEEV McM-derived fragments; black  =  WEEV IMP-derived fragments). Chimeric viruses were rescued from cells electroporated with infectious clones. Mosquito infectivity was determined by the presence of replication competent virus in the body 8 days after giving an infectious bloodmeal. Mice were infected by subcutaneous injection of 10^3^ pfu in 50 uL inoculum inside the right thigh. Mice were followed for 14 days after infection for signs of neurological disease. Unique, conserved restriction sites used for chimera clone construction along with their position within the WEEV McM genome are noted.

A panel of McM/IMP chimeras was constructed to identify the determinants of WEEV McM virulence in CD-1 mice (figure 1). Rescued virus was administered by subcutaneous inoculation of 6 week-old CD-1 mice. Chimera pair 1 containing exchanges in the E1 gene region retained phenotypes similar to the parental viruses with 86% mortality and 0% mortality for the McM-based and IMP-based chimeras, respectively. Exchange of the region from the unique AvrII restriction site at McM nt 7576 to the unique KpnI site at McM nt 9552 (pair 2; figure 1) did transfer the virulent phenotype with the McM-based chimera losing virulence and the IMP-based chimera becoming virulent (80%) but with a relatively delayed mean time to death of 7.9 days. These results suggested that virulence is determined by capsid, E3, and/or E2 genes.

Chimera pair 3 and 4 exchanged the capsid gene from the AvrII site to the 3′ end and the E2 gene from the 5′ terminus to the KpnI site (figure 1). Exchanging the capsid gene did not confer or remove the virulent phenotype. The IMP and McM-based chimeras exhibited 0% and 80% mortality in CD-1 mice, respectively. However, exchanging the E2 gene did have an effect on virulence. The McM-based chimera containing the IMP E2 gene completely lost its virulence while the IMP-based chimera with the McM E2 gene exhibited 40% mortality (MTD = 7.7 days). The E2 gene was further manipulated by exchanging E2 nts 1–518 or E2 nts 519–1226. Pair 5, exchanging E2 nts 1–518 retained the phenotype of the parental viruses, though mortality was further decreased for the McM-based chimera (30%). Placing the IMP-derived E2 nts 519–1226 in the McM backbone reduced mortality to 0% (pair 6, figure 1). The reciprocal chimera also exhibited reduced mortality (10%). Therefore the minimal necessary determinant essential for mouse virulence was narrowed to a region of McM E2 from nt 519 to the KpnI site at 1226.

**Figure 2:**
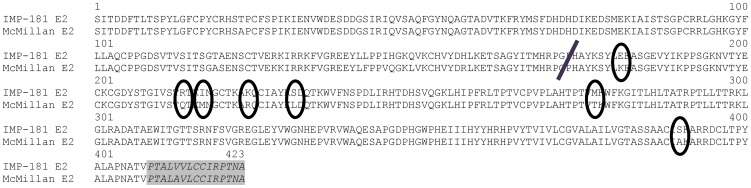
Amino acid alignment of WEEV McM and WEEV IMP E2 protein (accession numbers GQ287640 and GQ287641, respectively). The region shaded in gray is 3′ of the unique KpnI restriction site and not included in exchanges using this site. The mid-E2 cloning junction used in pairs 5 and 6 is denoted by the black bar (E2 nt 517, aa 173). The seven amino acid differences in the E2 region exchanged by pair 6 are circled.

Alignment of the IMP and McM E2 sequences (accession numbers GQ287641 and GQ287640, respectively) revealed 19 nucleotide differences between the strains in this region. Of these, 11 were silent and eight led to seven amino acid differences (two nucleotide changes are involved in a single amino acid change; figure 2). Fourteen point mutants were constructed representing the reciprocal chimeras for each of the seven loci. Exchanges at six of the seven loci (aa 181, 217, 224, 231, 277, and 390) retained the parental neurovirulence phenotype in the mouse. Mortality rates associated with the McM-based point mutants ranged from 70–100% (table 1). Despite retaining a generally virulent phenotype, placement of the IMP-like amino acid at positions 181 and 224 yielded significantly less mortality than the McM parent (p<0.05). Mortality among IMP-based mutants ranged from 0–10%, none significantly different than the IMP parent. Only the exchange of amino acid 214 (Q to R) affected the pathogenic phenotype. Mortality following infection with the IMP-based virus containing the McM E2 Q214 (IMP E2 R214Q) residue remained at the previously observed 0%. The reciprocal chimera [McM-based mutant containing IMP-like E2 R214 (McM E2 Q214R)] showed significant loss of virulence relative to the McM parent virus as only a single animal succumbed to disease (n = 10; p<0.001) indicating that while not sufficient to confer virulence in an otherwise attenuated backbone, the McM E2 amino acid 214 is necessary for virulence in our mouse model of WEEV infection. The single animal that succumbed following infection with the McM E2 Q214R appeared to follow an atypical disease course (hind limb paralysis only) that will be examined in future studies. That animal and the single mouse that succumbed to infection with IMP E2 S390A are the only examples in these studies of infection with a virus containing arginine at E2 aa 214 resulting in a lethal outcome.

**Table 1:** Mosquito infectivity in *Cx. tarsalis* and mouse mortality for E2 point mutants.

Mutant				
Strain	E2 aa position	Δ	% mosquito Infection (n)	% mouse mortality (MTD)*
McM	181	K-E	13.3 (30)	70 (6.7)
IMP	181	E-K	5.1 (32)	0
McM	214	Q-R	0(34)	10 (5)
IMP	214	R-Q	11.7(70)	0
McM	217	M-I	20.0 (30)	90 (4.4)
IMP	217	I-M	68.4 (19)	0
McM	224	R-K	37.1 (35)	80 (5.0)
IMP	224	K-R	72.4 (29)	0
McM	231	L-S	29.4 (34)	90 (4.6)
IMP	231	S-L	41.9(31)	0
McM	277	T-M	35.3(34)	100 (4.3)
IMP	277	M-T	54.5(33)	0
McM	390	A-S	29.6(27)	100 (4.5)
IMP	390	S-A	61.5(33)	10 (12.0)

* MTD = mean time to death in days post-infection.

### Baby chicken infection results with structural chimeras and E2 point mutations

**Figure 3:**
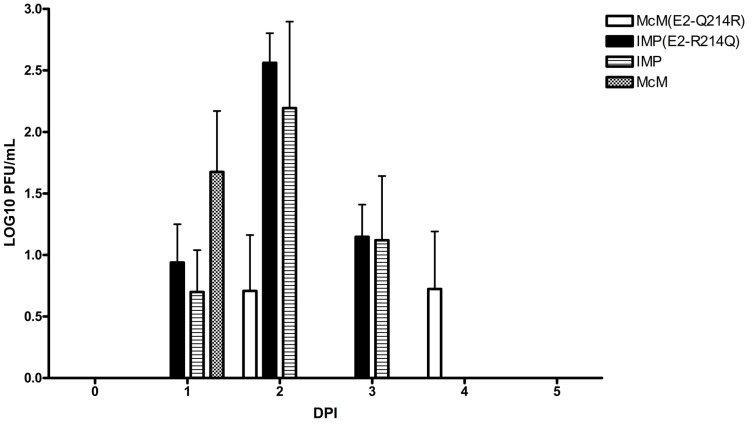
Viremia in 3-day-old chickens following subcutaneous challenge. Absence of bar indicates for all determinations viremia was below the limit of detection (0.82 log10 pfu/mL).

As avians are the natural reservoirs for WEEV, we further sought to examine the characteristics of these viruses in a readily available avian model. 10^4^ pfu of McM, IMP, McM E2 Q214R and IMP E2 R214Q viruses were subcutaneously injected into 3 day old chicks to observe any differences among the viruses in their resulting viremia within an avian model. Viremia in chicks was detected for McM at 1 dpi but no viremia was detected at days 2–3 dpi (figure 3). Viremia was detected at 1–3 dpi in chicks infected with IMP or IMP E2 R214Q virus. Though none of the viremia differences reached statistical significance, the highest measurable viremias occurred on day two (figure 3) and were associated with IMP or IMP E2 R214Q viruses. In the present study, none of the infected chicks showed signs of disease during the 7 dpi monitoring period. Hardy et al showed 1 day old chicks were highly susceptible to WEEV-induced disease [14]. It is not clear if the different results are due to an age-dependent susceptibility to WEEV disease or some other detail of our model. However, that viremia was more often associated with IMP infection suggests that the observed McM virulence in mice is not due to an extraordinary general virulence in vertebrates. Additional studies in avian hosts, including passerine birds, are proposed for the future.

### Mosquito infection results with structural chimeras and E2 point mutations

In addition to the mouse virulence phenotype, we used McM/IMP chimeras to investigate the determinants of the observed difference in mosquito infectivity. *Cx. tarsalis* mosquitoes were allowed to feed on blood meals containing each of the 12 structural chimeras and 14 E2 point mutation exchanges (figure 1, table 1). The infectivity of the parent strains was 10.0% and 78.9% for McM and IMP, respectively. Clone pairs 1 and 3 yielded no significant change from the original parent infection phenotype; however pair 2 restored the infection phenotype and suggested a determinant in the capsid, E3, or E2 regions. 87.9% of *Cx. tarsalis* given McM backbone virus were infected and 30% of mosquitoes given IMP backbone virus were infected. Clone pairs 4, 5, and 6 further identified the determinant of infection as residing in the same region of E2 (nts 519–1226) that determined mouse virulence. Pair 6 had a demonstrated infectivity of 23.8% for both rescued viruses, representing a deviation from the parental infection phenotype.

Infectivity for the point mutations at E2 amino acids 181 and 214 were 5.1% and 11.7%, respectively, for the IMP strain mutants and 13.3% and 0% for the McM mutants, representing a significant reduction of the IMP parental mosquito infection phenotype (p<0.001 for both IMP-based viruses), but not restoration (Table 1). The remaining pairs resulted in a slight reduction and an approximately 3-fold increase in infectivity for the IMP and McM-based chimeras, respectively (the pairs exchanging amino acid 224 and 231 will all significantly different than the parent viruses, p<0.05). These results suggest that while there may be multiple determinants, the IMP arginine at E2 position 214 is critical but not sufficient for mosquito infectivity.

### Multi-step growth curves of McM, IMP, and E2 aa 214 point chimeras

**Figure 4:**
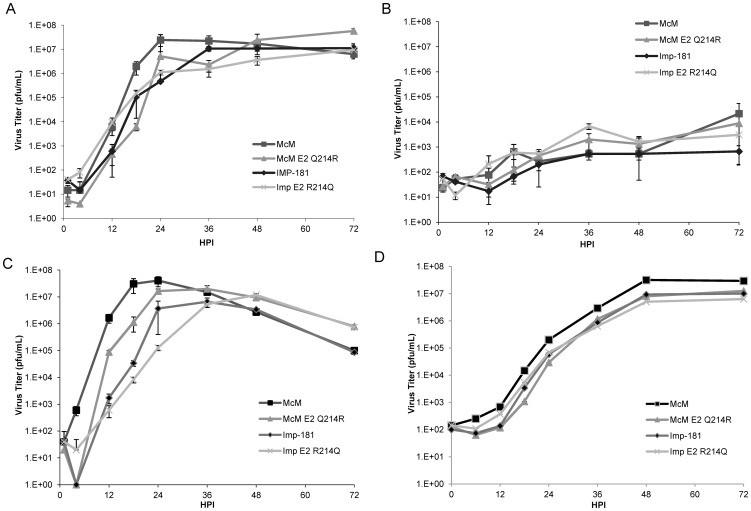
Multistep growth curves for WEEV strains McM and IMP, and WEEV clones McM E2Q214R and IMP E2R214Q in (A) BHK, (B) N2a, (C) DF1, and (D) C6/36 cells. 90% confluent cell monolayers in 24 well plates were infected at low multiplicity (MOI 0.01 to 0.05).

To determine whether the observed phenotypes were due to general replicative differences, multi-step growth curves of McM, IMP, or the McM or IMP-based E2 aa 214 point chimeras were performed in both mammalian and insect cells. In BHK cells, all four viruses grew similarly well, rapidly increasing in titer from 10–100 pfu/mL to 10^6^–10^7^ pfu/mL (figure 4A). No consistent statistical differences were observed among the four viruses, though the titer of McM E2 Q214R was significantly greater than McM, IMP, and IMP E2 R214Q 72 hpi (p<0.05).

N2a murine neuroblastoma cells were used to determine whether growth differences among viruses might account for the observed differences in mouse mortality due to neuron infection. Again, no overt growth difference was observed among, McM, IMP, McM E2 Q214R, and IMP E2 R214Q viruses through 72 hpi (figure 4B). Though at 72 hpi, IMP trended toward having lower virus titers than McM, at both 48 and 72 hpi, no statistically significant difference in virus titer was observed between any of the four viruses.

Cultured DF-1 chicken embryo fibroblast cells were infected with McM, IMP, McM E2 Q214R, and IMP E2 R214Q viruses to observe any growth differences that would be regulated by the virus-vertebrate reservoir dynamics. In this cell line, significant growth differences were observed, especially through 24 hpi (figure 4C). All 4 viruses were different at 18 hpi, with the McM and McM E2 Q214R McM viruses 1–2.5 logs higher than IMP and IMP E2 R214Q viruses at 24 hpi. Peak viral titers were significantly different between McM (24 hpi) and IMP (36 hpi), but no other viruses. The titers for the point mutants were significantly higher than the parent viruses at 48 and 72 hpi.

In C6/36 mosquito cells, all four viruses showed similar replication kinetics with no statistically significant differences observed (figure 4D). Mean titer measurements at 72 hpi ranged from 6.8 Log10 pfu/mL for IMP (E2-R214Q) to 7.5 Log10 pfu/mL for McM.

## Discussion

Extensive pathogenic studies have been performed on several alphaviruses strains and the availability of infectious clones for many alphaviruses has allowed reverse genetics studies to identify the molecular basis of various phenotypes, including vertebrate virulence and vector adaptation. In different models nsP2, nsP3, capsid, E1, and E2 were all identified as pathogenic determinants [2], [15]–[17].

In this study, we identified minimal infection and virulence determinants of two distinct WEEV phenotypes. A single amino acid, E2 Q214, was identified as necessary for WEEV McM-mediated pathogenesis in a murine model of infection. Importantly, the same amino acid position, E2 R214, was identified as necessary for efficient WEEV IMP-mediated infectivity of the primary mosquito vector, *Cx. tarsalis*. That these two phenotypes are determined by different amino acids at the same position suggests that lethality and transmission are mutually exclusive in this model, suggesting an explanation as to why there are so few mammalian cases in the presence of active transmission. Although IMP E2 R214Q virus had viremic profiles similar to IMP virus infected chicks, the R to Q change significantly affected mosquito infectivity. Curiously, in neither case did the reciprocal point chimera confer the examined phenotype to the previously incompetent parental virus. Such a transfer of increased virulence phenotype likely requires multiple loci, a notion supported by the variable mouse mortality among the chimeric viruses in this study.

Our *in vitro* studies conclude that the recombinant viruses used in this report do not have defects in replication as indicated by the growth curve analysis in multiple cell lines. In general, McM showed the most rapid replication kinetics compared to all other viruses. However, *in vitro* results did not correlate well with *in vivo* findings. Mouse virulence or mosquito infectivity of each virus, including the single point mutants, did not correlate with any differences in growth kinetics *in vitro*. These findings underscore the limitations of *in vitro* assays in predicting virulence in a mouse model of infection or infectivity of the primary vector mosquito species. *In vitro* studies remain necessary, however, to demonstrate that recombinant viruses are not overtly compromised with respect to replication kinetics.

Among alphaviruses, relatively little is known about WEEV structure and pathogenesis. Much must be extrapolated from studies of other alphaviruses, including Sindbis virus (SINV). The region of the SINV E2 glycoprotein from approximately amino acid 172 through 220 has been implicated in cell recognition [18], [19] and roughly corresponds to E2 domain B, a disordered domain from amino acid 167 through 254 [20]. Cryo-electron microscopic (cryo-EM) reconstruction of SINV E1-E2 complexes suggests that E2 amino acid 214 is located on the extreme tip of the E2 spike and within domain B [20], [21] supporting a direct role for E2 amino acid 214 in cell recognition or receptor binding. The exchange of a polar neutral glutamine for a positively charged arginine, and vice-versa, at position 214 could be expected to significantly alter such molecular interactions. Similarly, E2 amino acid 181, also involved in vector infectivity is likely located in domain B near the tip of E2. The amino acid switch at this position is more significant, exchanging positively charged lysine and negatively charged glutamic acid. This major change could affect interactions with cellular recognition molecules directly or indirectly by altering the conformation of the E2 spike tip.

Previous studies examining SINV vector adaptation have primarily implicated residues located in domain A. Pierro et al. found E2 amino acids 55, 70, 95, 96, and 116–118 to be involved in enhanced SINV infectivity of *Aedes* (*Ae.*) *aegypti* [22], [23]. While the mechanism is likely different based on the location within the protein, amino acids 55 and 70 present a situation analogous to the current report. SINV E2 Q55H/E70K results in increased virulence in suckling mice and increased binding to neuronal cells [24], [25]. Viruses containing the reciprocal mutations, E2 H55Q/K70E demonstrate increased infectivity for *Ae. aegypti* [22]. Chikungunya E2 amino acid 211 and Venezuelan equine encephalitis virus E2 amino acids 207, 213, and 218 were implicated in adaptation to their respective vectors [26]–[28]. X-ray crystallographic and cryo-EM structures also place these amino acids at or near the tip of the E2 spike [29], [30].

Nagata et al. previously examined the molecular determinants of WEEV pathogenesis in mice [31]. That study differed from the current study in several ways: 7–20 week old BALB/c mice were used, mice were inoculated by the intranasal route, and all isolates used were 100% lethal to mice in this model. The authors showed by correlating structural protein phylogenetic analysis and virulence that the three most virulent strains clustered together and the five least virulent strains clustered together with sequence divergence apparently correlating with changing virulence. Interestingly, two of the three most virulent strains contain E2 Q214. One of these two strains is McM and the other California, a strain phylogenetically closely related to McM. The remaining strains, including Fleming, the most virulent strain used in that study, contain IMP-like E2 R214. That five different uniformly lethal strains contain E2 R214 is not surprising. Given that our data suggest the presence of multiple loci responsible for pathogenesis, it is possible that other differences between these five strains and the McM/California genotype compensate for R214 to render them pathogenic to mice that is not present in IMP or McM. Additional studies will be required to address this possibility.

In summary, we have used our previously defined mouse model of WEEV pathogenesis to determine that different amino acids at the same position within the E2 glycoprotein are necessary for both WEEV McM mouse virulence and WEEV IMP vector infectivity. WEEV IMP may have an advantage in the transmission cycle by efficiently infecting the avian host and producing high vector competence in the *Cx. tarsalis* strain. Our studies indicate that increased virulence in CD-1 mice is accompanied by decreased mosquito infectivity, suggesting a plausible explanation for the current lack of epidemic WEEV.
